# An Anthracene Carboxamide-Based Fluorescent Probe for Rapid and Sensitive Detection of Mitochondrial Hypochlorite in Living Cells

**DOI:** 10.3390/bios13090883

**Published:** 2023-09-12

**Authors:** Xueling Liu, Yali Wang, Guangshuai Zhou, Wenzhou Zhang

**Affiliations:** 1 Department of Pharmacy, The Affiliated Cancer Hospital of Zhengzhou University & Henan Cancer Hospital, Zhengzhou 450008, China; liuxueling4718@zzu.edu.cn; 2 School of Pharmaceutical Science and Technology, Health Sciences Platform, Tianjin University, Tianjin 300072, China; 3 Department of Chemistry, College of Pharmacy, North China University of Science and Technology, Tangshan 063000, China

**Keywords:** hypochlorite, mitochondria, anthracene carboxyimide, fluorescent probe

## Abstract

Mitochondrial hypochlorite (ClO^−^) plays important and often contradictory roles in maintaining the redox balance of mitochondria. Abnormal ClO^−^ levels can induce mitochondrial inactivation and further cause cell apoptosis. Herein, we have developed an anthracene carboxyimide-based fluorescent probe **mito-ACS** for imaging mitochondrial ClO^−^ in living cells. This probe exhibits some distinctive features as excellent resistance to photobleaching, high selectivity and sensitivity, as well as good water solubility. **Mito-ACS** showed a noticeable fluorescence response toward ClO^−^ with a fast response (within 6 s) and a low detection limit (23 nM). Moreover, the introduction of triphenylphosphonium makes the probe soluble in water and selectively localizes to mitochondria. Furthermore, **mito-ACS** was successfully applied to image mitochondria ClO^−^ in living cells with low toxicity. Remarkably. the less used fluorophore anthracene carboxyimide exhibiting excellent photostability and desirable optical properties provides a promising application prospect in biological systems.

## 1. Introduction

Hypochlorite/hypochlorous acid (ClO^−^/HOCl), one of the most important reactive oxygen species (ROS), plays vital roles in biological activities and daily life [1,2,3]. ClO^−^ is commonly used as a household bleach and a disinfectant [4]. More importantly, ClO^−^ is linked to diverse physiological and pathological functions, especially in the immune defense system [5,6,7]. However, the abnormal production of ClO^−^ can also result in tissue damage and remodeling with profound implications for many human diseases such as arthritis, cardiovascular disease, and even cancer [8,9,10,11]. The mitochondria, as an “energy factory”, is considered the main source of ROS in cells including ClO^−^ [12,13]. It has been reported that abnormal ClO^−^ can destroy the mitochondrial permeability transport pores and apoptosis signal molecules, leading to a nonspecific increase in membrane permeability and cell damage [14,15]. Therefore, exploring reliable methods for monitoring ClO^−^ in mitochondria is still in high demand.

The results of nearly a decade have amply demonstrated the advantages and practical applications of fluorescent probes for the detection of reactive oxygen species [16,17,18]. Also, with the development of fluorescence detection systems, fluorescence analysis is more likely to become the first choice of commonly used detection. More importantly, the development of organelle-targeted fluorescent probes made the precise monitoring of subcellular microenvironments come true [19,20,21]. To date, a large number of hypochlorite probes have been reported and applied in the biosystem. Each probe indeed possessed its advantages and drawbacks [22,23,24,25]. It has been proved that the following points are relevant to the ability of the probe to detect hypochlorite in living organisms and be used for biological imaging. A key issue is the sensitivity. Due to the low concentration in living organisms, the probe must respond rapidly to ClO^−^ and has a low detection limit [26,27,28]. Another fact is that the probes with good photostability are preferred in the bioimaging application, which can minimize the impact of photobleaching and extend the optical monitoring [29,30]. However, to our knowledge, there are few probes with both characteristics. Hence, developing probes with substantial photostability, sensitivity, and selectivity for monitoring mitochondrial ClO^−^ becomes our target.

**Figure 1:**
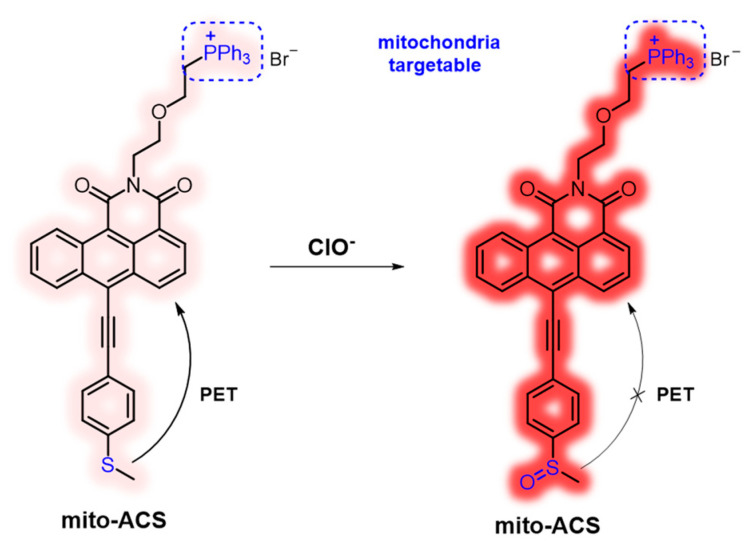
Design strategy of **mito-ACS** for sensing ClO^−^.

In this work, a new mitochondria-targeted fluorescent probe **mito-ACS** was rationally designed through the integration of ClO^−^-sensing thioether, mitochondria-targeting triphenylphosphonium cation, and recently developed fluorophore anthracene carboxamide as shown in Figure 1. The construction of **mito-ACS** was on the following facts: first, 1,2-anthracenecarboximide derivatives, similar but prior to 1,8 naphthalimide in many photoproperties including good photostability, visible absorption and emission with a large Stokes shift, and high quantum yield [31,32,33]. Also, the absorption and fluorescence spectra of anthracenecarboximide could be easily modulated by varying the electron-donating capability of the substituent at the 6-position. Secondly, many studies, including our previous work, have shown that thioethers exhibit high reaction efficiency and selectivity to hypochlorite [22,34]. The thioether with strong electron-donating ability can quench the emission of **mito-ACS** through an effective (PET) process [35,36,37], and the probe would recover its strong fluorescence once the PET process is blocked by the ClO^−^-oxidized product sulfoxide. In addition, the introduction of acetylene can obtain a relatively longer analytical wavelength by extending the conjugated length of the aromatic donor part. Third, triphenylphosphine, as a mitochondrial targeting group, can also increase the water solubility of **mito-ACS** [21]. Based on the principles, **mito-ACS** exhibited multiple advantages, such as excellent photostability (more than 80 min), large Stokes shift (~100 nm), high sensitivity (detection limit of 23 nM), and fast response (within 6 S) toward ClO^−^. Moreover, **mito-ACS** showed good solubility in water, which enables it to be successfully applied to real-time detection of mitochondria ClO^−^ in biological imaging.

## 2. Materials and Methods

### 2.1. Reagents and Instruments

All reagents used in the reaction were purchased from commercial suppliers without further purification unless otherwise stated. The ^1^H NMR and ^13^C NMR spectra were measured by Bruker Ascend ΙΙΙ 400 and Avance ΙΙΙ 600 instruments at 298.6 K using CDCl_3_ and DMSO (d_6_) as solvents. ESI-MS data were collected on Agilent Technologies 6230 TOF LC/MS with ESI source. The UV and fluorescent measurements were performed on a HITACHI U-3900 spectrophotometer and an Edinburgh FLS980 spectrophotometer, respectively. The photodynamic experiment was carried out using an FLS980 with a 450 W xenon lamp. The absolute quantum yield method was carried out using an integrating sphere detector from Edinburgh Instruments. The ultrapure water was obtained by the Direct-Q5 purification instrument. The analysis for the reaction mixture of **mito-ACS** with NaClO was performed by using a Shimadzu LC-20A HPLC with a C-18 reversed-phase column. A Leica SP 8 confocal laser scanning microscope was used for fluorescence imaging. HeLa Cells were purchased from the Type Culture Collection of the Chinese Academy of Sciences, Shanghai, China.

### 2.2. Synthesis

**Scheme 1:**
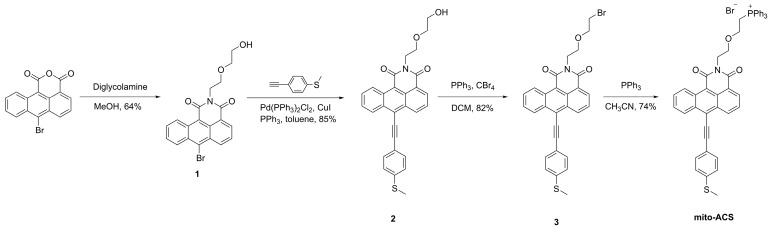
Synthetic route of **mito-ACS.**

The anthracene carboxyimide-based probe **mito-ACS** was synthesized in Scheme 1. The synthesis of starting material 6-bromo-1,2-anthracene dicarboxylic acid anhydride is shown in the Supporting Information. Intermediate **1** and 1-Ethynyl-4-(methylthio)benzene were synthesized according to the reported procedures [38,39]. Compound **2** was obtained through a Suzuki cross-coupling reaction in 85% yield; then, it was converted to the bromide adduct **3** through an Apple reaction in 82% yield. Finally, the desired product **mito-ACS** was obtained by treating intermediate **3** with triphenylphosphine in 74% yield. The detailed experimental are as follows and the characterization spectra are shown in the Supporting Information.

The synthesis of compound **1**

6. -Bromo-1,2-anthracene dicarboxylic acid anhydride (330 mg, 1 mmol) and 2-(2-aminoethoxy)ethanol (150 uL, 1.5 mmol) were dissolved in methanol (10 mL) under a nitrogen atmosphere. The reaction mixture was stirred under 60 °C for 4 h. Once the TLC plate showed that all the starting material was consumed, the reaction mixture was cooled to room temperature and concentrated under vacuum. Then, water (50 mL) was added and extracted by dichloromethane (20 mL × 3), and the organic phase was collected and washed with brine (100 mL), dried over Na_2_SO_4_, and concentrated via rotary evaporator. The crude product was purified by column chromatography (CH_2_Cl_2_) to give the product 1 as a yellow solid (64%). ^1^H NMR (400 MHz, CDCl_3_) δ 9.99 (d = 9.1 Hz, 1H), 8.87 (d, *J* = 8.8 Hz, 1H), 8.76 (d, *J* = 7.0 Hz, 1H), 8.65 (d, *J* = 8.8 Hz, 1H), 7.88–7.74 (m, 2H), 7.70 (t, *J* = 8 Hz), 4.53 (t, *J* = 5.7 Hz, 2H), 3.92 (t, *J* = 5.7 Hz, 2H), 3.71 (m, 4H). ^13^C NMR (151 MHz, CDCl_3_) δ 164.9, 163.4, 135.1, 134.6, 134.0, 134.0, 131.4, 131.4, 129.0, 128.6, 128.4, 128.1, 127.0, 126.8, 122.5, 115.2, 72.3, 68.5, 61.9, 39.9.

The synthesis of compound **2**

To a mixture of toluene (10 mL), CuI (30 mg, 0.15 mmol) and PPh_3_ (26 mg, 0.1 mmol), solution of **1** (413 mg, 1 mmol), and 1-ethynyl-4-(methylthio)benzene (180 mg, 1.2 mmol) were added, and then the mixture was degassed 3 times by evacuating the flask and backfilling of Ar. Then, PdCl_2_(dppf) (35 mg, 0.05 mmol) was added, and the mixture was stirred at 110 °C for 12 h. The reaction mixture was cooled to RT, diluted with dichloromethane (15 mL), and filtered through Celite. The filtrate was extracted with dichloromethane (3 × 20 mL). The combined organic phases were washed with brine (40 mL) and concentrated under reduced pressure. The residue was purified by column chromatography with CH_2_Cl_2_:MeOH (*v*/*v*, 100/1) to afford **2** as a yellow solid (85%). ^1^H NMR (600 MHz, CDCl_3_) δ 9.69–9.47 (d, *J* = 6.1, 1H), 8.33 (m, 2H), 8.13 (s, 1H), 7.49 (s, 1H), 7.41–7.24 (m, 4H), 7.14 (d, *J* = 6.2 Hz, 2H), 4.30 (m, 2H), 3.80 (m, 2H), 3.66 (m, 4H), 2.47 (s, 3H).^13^C NMR (151 MHz, CDCl_3_) δ 164.47, 163.53, 141.68, 133.28, 132.47, 132.09, 130.82, 128.85, 127.39, 127.30, 127.27, 126.89, 126.77, 125.67, 125.58, 122.09, 118.19, 114.03, 106.43, 85.74, 72.36, 68.52, 61.89, 39.70, 15.13.

The synthesis of compound **3**

Compound **2** (240 mg, 0.5 mmol), triphenylphosphine (200 mg, 0.75 mmol), and carbon tetrabromide (245 mg, 0.75 mmol) were added to 20 mL of dichloromethane. The solution was stirred for 24 h at room temperature. Then, water was added and extracted with dichloromethane (3 × 20 mL). The organic layer was collected and dried over brine (30 mL), anhydrous Na_2_SO_4_, and then, concentrated via rotary evaporator. The crude product was purified by column chromatography CH_2_Cl_2_:MeOH (*v*/*v*, 100/1) and then washed by n-hexane to give the product **3** as a yellow solid (82%). ^1^H NMR (400 MHz, CDCl_3_) δ 9.91 (d, *J* = 9.1 Hz, 1H), 8.78 (d, *J* = 9.0 Hz, 1H), 8.67 (d, *J* = 6.9 Hz, 1H), 8.59 (d, *J* = 8.6 Hz, 1H), 7.79–7.72 (m, 1H), 7.64 (m, 4H), 7.30 (d, *J* = 8.3 Hz, 2H), 4.50 (t, *J* = 6.1 Hz, 2H), 3.90 (dt, *J* = 8.3, 6.2 Hz, 4H), 3.46 (t, *J* = 6.2 Hz, 2H), 2.57 (s, 3H). ^13^C NMR (101 MHz, CDCl_3_) δ 164.68, 163.55, 141.75, 133.67, 133.47, 132.90, 132.61, 132.17, 131.02, 129.39, 127.78, 127.60, 127.16, 127.07, 125.91, 125.77, 122.51, 118.23, 114.62, 106.46, 85.85, 70.63, 68.02, 39.26, 31.95, 15.18.

The synthesis of probe **mito-ACS**

Compound **3** (160 mg, 0.3 mmol) and triphenylphosphine (105 mg, 0.4 mmol) were added to 8 mL of acetonitrile. The solution was refluxed for 3 h, and then, after being cooled to room temperature, the mixture was concentrated via rotary evaporator. The crude product was purified by column chromatography CH_2_Cl_2_:MeOH (*v*/*v*, 20/1) to provide the product **mito-ACS** as a red solid (74%). ^1^H NMR (600 MHz, CDCl_3_) δ 9.75 (d, *J* = 9.0 Hz, 1H), 8.72 (d, *J* = 8.4 Hz, 1H), 8.52 (d, *J* = 6.6 Hz, 2H), 7.75 (dd, *J* = 12.8, 7.7 Hz, 6H), 7.72–7.67 (m, 1H), 7.66–7.50 (m, 13H), 7.27 (d, *J* = 8.2 Hz, 2H), 4.18 (m, 2H), 4.12 (t, *J* = 6.0 Hz, 2H), 4.03 (t, *J* = 5.5 Hz, 1H), 3.99 (t, *J* = 5.5 Hz, 1H), 3.51 (t, *J* = 6.0 Hz, 2H), 2.55 (s, 3H). ^13^C NMR (151 MHz, CDCl_3_) δ 163.29, 162.16, 141.01, 133.57, 133.55, 133.00, 132.93, 132.75, 132.39, 131.77, 131.48, 131.19, 130.12, 129.07, 128.98, 128.24, 126.80, 126.63, 126.24, 125.82, 124.91, 124.82, 121.22, 118.20, 117.63, 117.05, 113.16, 103.94, 84.75, 67.14, 63.23, 63.17, 37.96, 24.61, 24.26, 14.18. ESI-MS: m/z calculated for C_47_H_37_NO_3_PS^+^ [M]^+^ 726.2226, found 726.20.

### 2.3. Preparation of the Spectral Measurements

The **mito-ACS** stock solution with a concentration of 1 mM was prepared with deionized water. An amount of 20 μL of the probe was diluted with PBS buffer (10 mM, pH = 7.4), and different equivalents of ClO^−^-stock solution were added according to the experimental requirements. The final volume was 2 mL by PBS buffer, and the final concentration of the probe was 10 μM. Homogeneous mixing and reaction were continued for 5 min after the relevant spectral test. The same method was also applicable to selective experiments. The method for the photodynamic experiment was that 20 μL of the probe was diluted with PBS buffer (10 mM, pH = 7.4, 1960 μL), and 20 μL of ClO^−^-stock solution (1 mM and 2 mM) was added. When mixing well and measuring immediately, the time-dependent fluorescence intensity changes at 575 nm. The method of configuring different pH buffers was achieved by adding a minimum volume of NaOH (0.2 M) or HCl (0.2 M) to PBS (10 mM, pH = 7.4). The fluorescence spectrum test conditions were an excitation wavelength of 480 nm and a slit of 2 nm.

### 2.4. Cell Viability Assay

HeLa cells were incubated in Dulbeccos modified Eagles medium (DMEM) supplement with 10% (*v*/*v*) fetal bovine serum (FBS, Gibco), 100 U/mL penicillin, and 100 μg/mL streptomycin at 37 °C with 5% CO_2_ in appropriate humidity. Logarithmic phase cells were digested with pancreatic enzymes, and cell suspension for implantation into a 96-well plate (10^4^ cells and 200 μL DMEM/per well) was prepared. After incubation for 24 h, the culture medium was removed and a new medium containing different concentrations of probe **mito-ACS** (0, 5, 10, 15, 20 μM) was added for another 24 h. The experimental group with a probe concentration of 0 served as a control. After that, 20 μL CCK-8 was added to each well and incubated for 4 h. The absorbance was measured by a microplate reader at 450 nm.

### 2.5. Confocal Fluorescence Imaging

Confocal fluorescence imaging was collected on a Leica SP 8 confocal laser scanning microscope. To investigate the response ability of **mito-ACS** to ClO^−^ in vivo, HeLa cells were divided into 4 groups. The control group of HeLa cells was treated with probe **mito-ACS** (5 μM) for 30 min, and a parallel group of Hela cells was pretreated with N-acetylcysteine (NAC, 500 μM) for 2 h and then treated with NaClO (30 μM). To detect the exogenous ClO^−^, the third group of HeLa cells was treated with **mito-ACS** (5 μM) for 30 min, and then treated with NaClO (30 μM) for another 30 min. To detect the endogenous ClO^−^, the fourth group of HeLa cells was treated with Lipopolysaccharide (LPS, 1 μg/mL) and phorbol 12-myristate 13-acetate (PMA, 1 μg/mL) for 12 h, and then treated with probe **mito-ACS** (5 μM) for 30 min. The cells were washed with PBS (pH 7.4) three times before imaging, and fluorescence was collected in a red channel (580–620 nm) with the excitation wavelength at 488 nm. To determine the subcellular localization, **mito-ACS** (5 μM) and Mito-Tracker Green (200 nM) were co-incubated in cells for 30 min, and then washed with PBS three times. The fluorescence was collected in a green channel (λ_ex_ = 488 nm, λ_em_ = 500–520 nm) and a red channel (λ_ex_ = 488 nm, λ_em_ = 600–620 nm).

## 3. Results

**Figure 2:**
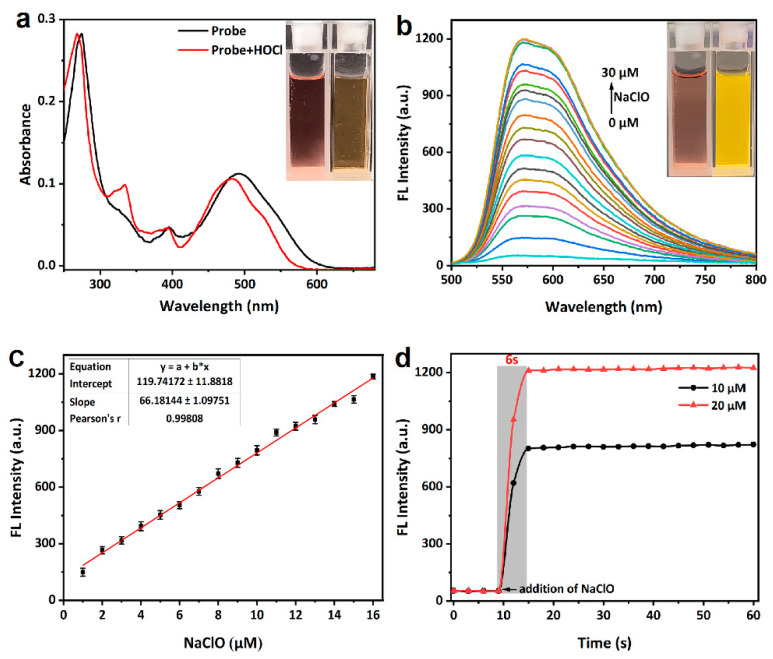
(**a**) UV-Vis absorption (10 μM) and (**b**) fluorescence spectra (10 μM) changes of **mito-ACS** upon addition of increasing amount of NaOCl (0–30 μM), (**c**) fluorescence intensity at 575 nm of **mito-ACS** versus concentrations of ClO^−^ (* represents multiplication), and (**d**) time-dependent fluorescence intensity changes of **mito-ACS** (10 μM) upon addition of ClO^−^ (10 μM/20 μM). (PBS, 10 mM, pH = 7.4), λ_exc_ = 480 nm, slits = 2/2 nm. Inset: (**a**) the color and (**b**) fluorescence images of **mito-ACS** in the absence/presence of ClO^−^.

The spectroscopic properties of probe **mito-ACS** and its response to ClO^−^ were first investigated in PBS buffer (10 mM, pH = 7.4). The probe itself was nearly nonfluorescent (Φ = 0.06), and the absorbance peak was located at 492 nm (ε = 11,250 M^−1^ cm^−1^, Figure 2a). Upon the addition of ClO^−^ (3 equiv.), the absorbance of the oxidation product displayed a blue-shift relative to **mito-ACS**: 479 nm (ε = 10,600 M^−1^ cm^−1^, Figure 2a) versus 492 nm, and a strong fluorescence emission at 575 nm appeared (Figure S1). The quantum yield of the probe’s oxidation product increased to 0.28, strongly suggesting the excellent responsiveness of the probe to hypochlorite. In addition, the oxidation product showed a large Stokes shift (96 nm), which minimized the background interference of the initial probe and achieved a clear separation of excitation and emission. Based on experimental results and previously reported work, it is known that the sulfide has the electron-donor ability to quench fluorescence through the photo-induced electron transfer (PET) mechanism, and, therefore, **mito-ACS** exhibited weak fluorescence. Upon the addition of ClO^−^, the sulfide was oxidized to sulfoxide, which blocked the PET process, resulting in a significant fluorescence enhancement. To confirm this conjecture, we checked the mixture of **mito-ACS** and ClO^−^ by ESI-MS. As shown in Figure S2a, the m/z peak of the oxidation product of **mito-ACS** was found as the main peak of 742.20, which was in agreement with the theoretical calculation values of [**mito-ACSO**]^+^: 742.2176. This result supported the formation of the sensing product, sulfoxide, from oxidation of the probes by ClO^−^. The sensing mechanism was further verified by HPLC. The results are shown in Figure S2b, the chromatographic peak of the probe **mito-ACS** appeared at about 4 min. With the addition of NaClO, the **mito-ACS** peak gradually weakened and disappeared, and a new chromatographic peak appeared and gradually enhanced with a retention time of about 2.8 min, which was attributed to the fluorescent product sulfoxide. Next, given the probe’s initial design, we evaluated the pH influence on **mito-ACS**. As shown in Figure S3, the probe showed weak and negligible fluorescence over a wide pH range of 3–10, indicating that the thioether was insensitive to environmental pH changes. Meanwhile, the strong fluorescence of the oxidation product remained stable at pH 3–9. Since the pH value of mitochondria is about 7.99, results indicate that **mito-ACS** could function properly in physiological conditions, including the mitochondria [40].

Then, titration experiments were carried out to explore **mito-ACS**’s response to various equivalents of ClO^−^ upon excitation at 480 nm (PBS, 10 mM, pH = 7.4). The fluorescence intensity increased gradually with the concentration range of NaClO from 0 μM to 30 μM (Figure 2b). Notably, the fluorescence intensity at 575 nm showed a good linear relationship (R^2^ = 0.995) with ClO^−^ over the concentration range of 0~16 μM (Figure 2c). The detection limit was calculated to be 23 nM from the formula S/N = 3 (see Supporting Materials for details) [41]. These results all indicated that the probe **mito-ACS** could be used for quantitative detection of ClO^−^. In addition, the response time of **mito-ACS** (10 μM) with ClO^−^ (10 μM/20 μM) was investigated in PBS solution (10 mM, pH = 7.4). As shown in Figure 2d, when different equivalents of ClO^−^ were added, the fluorescence intensity at 575 nm was rapidly enhanced, and both reached the maximum around 6 s Furthermore, the fluorescence signal of the oxidation product remained almost unchanged under continuous irradiation by a 450 W xenon lamp (Figure S4). Both the fast response time and high photostability are essential and suitable for the detection of ClO^−^ in vivo since the short-lived ClO^−^ and the complex biological environment.

**Figure 3:**
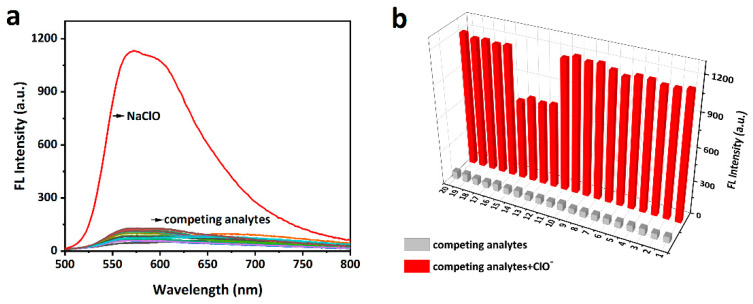
(**a**) Fluorescence emission spectra of **mito-ACS** (10 µM) with ClO^−^ (30 µM) and competing analytes (100 µM). (**b**) Fluorescence spectra response of **mito-ACS** (10 µM) with various analytes (100 µM) in response to ClO^−^ (30 µM). (1–20: H_2_O_2_, •OH, O_2_^−^, ROO•, NO•, TBHP, •OtBu, ONOO^−^, Ca^2+^, Mg^2+^, Cu^2+^, Fe^2+^, Hcy, Cys, GSH, Hg^2+^, Cu^+^, Fe^3+^, Zn^2+^, Al^3+^. (PBS, 10 mM, pH = 7.4), λ_exc_ = 480 nm, slits = 2/2 nm.

The selectivity of the probe (10 μM) toward ClO^−^ and other bio-related interfering species including ROS/RNS (H_2_O_2_, •OH, O_2_^−^, ^1^O_2_, ROO•, NO•, TBHP, •O*^t^*Bu, metal ions (Ca^2+^, Hg^2+^, Mg^2+^, Cu^2+^, Cu^+^, Zn^2+^, Fe^3+^, Fe^2+^, Ag^+^ Al^3+^), and biothiols (Cys, Hcy, and GSH) was verified in the PBS solution (10 mM, pH = 7.4) [42]. As shown in Figure 3a, only ClO^−^ induced a strong fluorescence enhancement (>50 fold) while other species led to negligible response. Notably, hydroxyl radical (•OH, highly reactive oxygen radical) and Hg^2+^ (highly reactive with a sulfur atom) did not noticeably enhance the fluorescence intensity of **mito-ACS**. In addition, the interference experiments were further studied by adding ClO^−^ (30 μM) to the probe in the presence of the competing species (100 μM), as shown in Figure 3b, **mito-ACS** displayed similar results in the fluorescence enhancement even in the presence of the interfering species. And lower fluorescence enhancements were observed in the presence of Fe^2+^ and biothiols (Cys, Hcy, and GSH). This could be attributed to their reducing properties reacting with ClO^−^, thereby consuming ClO^−^ to some extent, leading to the fluorescence intensity decrease. Nevertheless, the ClO^−^ still triggered obvious fluorescence enhancement (>20 fold) compared to the probe itself. The concentration of biothiols in organisms is about 1~10 mM. Therefore, to verify the robustness of the results, we examined the fluorescence spectral response of **mito-ACS** (20 μM) to ClO^−^ in the presence of high concentrations of GSH (5 mM). Results showed that the fluorescence intensity increased significantly even in the presence of physiological concentrations of GSH (Figure S5a). Meanwhile, the fluorescence intensity remained stable when physiological concentrations of GSH were added to the reaction solution of the probe **mito-ACS** with ClO^−^, indicating that the oxidation product, sulfoxide, was not affected by the reduction of GSH (Figure S5b). The above results indicated that **mito-ACS** exhibited excellent selectivity to ClO^−^ in the presence of potential biological interferences, further confirming the potential of the probe to detect ClO^−^ in complex biological systems.

**Figure 4:**
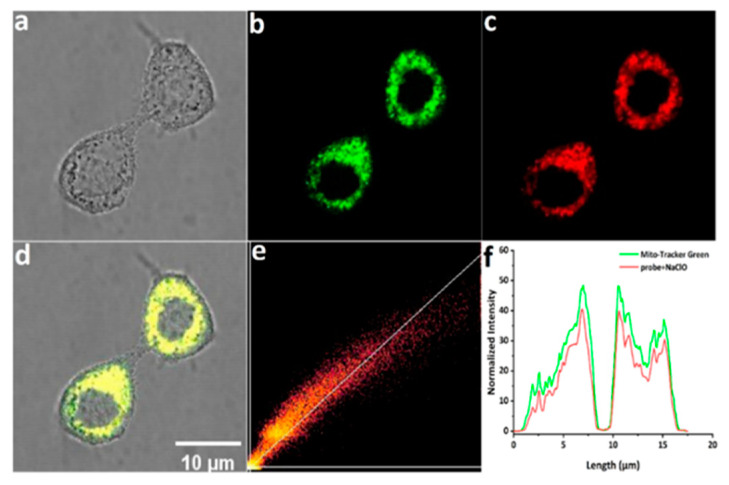
Confocal fluorescence images of living Hela cells incubated with **mito-ACS** (5 μM) and Mito-Tracker Green for 30 min: (**a**) bright-field imaging; (**b**) fluorescence imaging of Mito-Tracker Green (200 nM) in the green channel (λ_exc_ = 488 nm, λ_em_ = 500–520 nm); (**c**) fluorescence imaging of **mito-ACS** in the red channel (λ_exc_ = 488 nm, λ_em_ = 600–620 nm); (**d**) merged imaging of (**b**,**c**); (**e**) intensity correlation of **mito-ACS** and Mito-Tracker Green (**f**) intensity distribution of the linear region of living Hela cells co-stained with **mito-ACS** and Mito-Tracker Green. Scale bar: 10 μm.

Based on the excellent response of **mito-ACS** to hypochlorous acid in vitro, we further explored the capability of probes for mitochondrial ClO^−^-imaging in living Hela cells. Before imaging, the standard CCK-8 assay was conducted to evaluate the biocompatibility of **mito-ACS**. As shown in Figure S6, compared with the control group (cells without probe), the relative cell viability was up to 90% after incubation with 20 μM **mito-ACS** for 24 h, suggesting that the probe possesses negligible cytotoxicity and good biocompatibility. In the next step, the mitochondria-targeting ability was investigated with the colocalization experiment by co-incubating **mito-ACS** (5 μM) and the Mito-Tracker Green (200 nM, a commercial mitochondrial targeting dye). The green fluorescence from the Mito-Tracker Green and red fluorescence from **mito-ACS** treated with 30 μM ClO^−^ overlapped well (Figure 4a–d). Moreover, a high Pearson’s coefficient of 0.952 was obtained from the intensity correlation plots (Figure 4e), and the fluorescence intensity profile of regions of interest (ROI) across HeLa cells in two channels also varied synchronously (Figure 4f). Colocalization experiments showed that **mito-ACS** possessed good cell membrane permeability and accurate mitochondria-targeting ability. These properties could be attributed to the introduction of triphenylphosphonium, a function group that aggregated the probe in mitochondria and also increased the probe’s water solubility.

**Figure 5:**
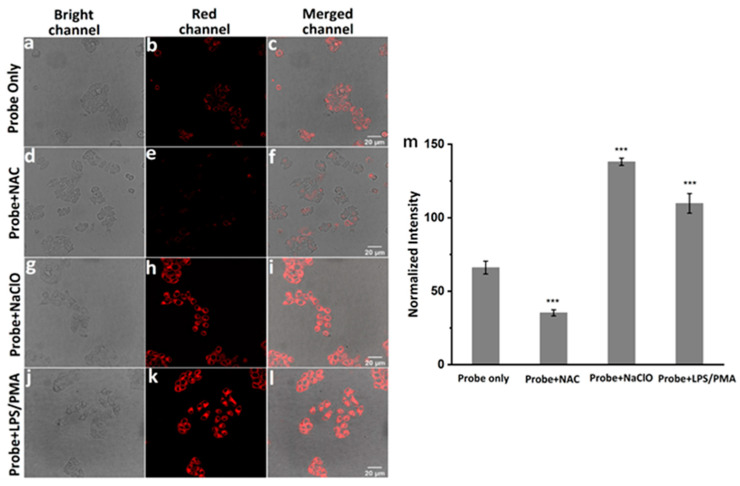
Confocal fluorescence images of living Hela cells incubated with (**a**–**c**) blank control, **mito-ACS** (5 μM) for 30 min; (**d**–**f**) NAC (500 μM) for 2 h, then **mito-ACS** (5 μM) for 30 min; (**g**–**i**) **mito-ACS** (5 μM) for 30 min, then NaClO (30 μM) for 30 min; (**j**–**l**) LPS and PMA (1.0 mg mL^−1^) for 2 h, then **mito-ACS** (5 μM) for 30 min. (**m**) Normalized fluorescence intensity of cells in panels (**b**–**k**) (n = 5, *** *p* < 0.001 versus controls). λ_exc_ = 488 nm, λ_em_ = 580–620 nm, Scale bars are 20 μm.

Finally, cell imaging experiments were carried out to evaluate the response ability of **moto-ACS** to ClO^−^ in living Hela cells. LPS and phorbol 12-myristate 13-acetate PMA are the known stimulants for cells to produce endogenous ClO^−^, while the NAC could remove the ROS including ClO^−^ [26,43]. Four groups of experiments were set as in the following: (1) incubation with probe (5 μM) for 30 min as control, (2) incubation with NAC (500 μM) for 2 h, then probe (5 μM) for 30 min, (3) incubation with probe (5 μM) for 30 min, then NaClO (30 μM), (4) incubation with LPS and PMA (1.0 mg mL^−1^) for 2 h, then probe (5 μM). As shown in Figure 4, the controlled Hela cells displayed a weak fluorescence (Figure 5a–c). Upon treatment with NAC, the fluorescence decreased significantly (Figure 5d–f), indicating that a small concentration of ClO^−^ exists in cells, and the probe exhibited high sensitivity to the variation of ClO^−^ concentration. As we expected, the fluorescence intensities were greatly enhanced under the LPS, PMA, and NaClO stimulation (Figure 5g–l). Comparing the fluorescence intensities from the four groups (Figure 5m), we concluded that **mito-ACS** could be stained in mitochondria accurately and was well-suited for monitoring exogenous and endogenous ClO^−^ inside living Hela cells. 

## 4. Conclusions

In summary, we have presented the design strategy, synthesis, and practical application of a new probe, **mito-ACS**, for ClO^−^ detection in pure aqueous media and living Hela cells. **Mito-ACS** contains an anthracene carboxyimide core with red emission and a hypochlorite-triggered fluorescence “off−on” switch-thioether that affords excellent sensitivity. Surprisingly, the introduction of triphenylphosphonium makes the probe completely soluble in water and selectively localizes to mitochondria. Spectrometric analysis showed **mito-ACS** possess desirable optical properties in imaging ClO^−^, such as high selectivity, fast response, and large Stokes shift. Furthermore, confocal fluorescence imaging revealed **mito-ACS** successfully achieved the monitoring of exogenous and endogenous mitochondrial ClO^−^ with low cytotoxicity. These features make **mito-ACS** uniquely suited for exploring ClO^−^ biology under a variety of physiological and pathological contexts.

## Data Availability

Not applicable.

## Supplementary Materials

The following supporting information can be downloaded at: https://www.mdpi.com/article/10.3390/bios13090883/s1, Figure S1: Fluorescence spectra of the probe **mito-ACS** (10 μM) with and without excessive ClO^−^ (30 μM) in pure aqueous media; Figure S2: MS-ESI and HPLC spectra of **mito-ACS** and the reaction mixture of **mito-ACS** with NaOCl; Figure S3: Fluorescence response of **mito-ACS** (10 μM) in the absence and presence of NaOCl (30 μM) at different pH solutions; Figure S4: Time-dependent fluorescence intensity changes of **mito-ACS** (10 μM) under irradiation with a 450 w lamp; Figure S5: Reactivity of probe **mito-ACS** and the oxidation product toward GSH; Figure S6: Cell viability of HeLa cells treated with different concentrations of **mito-ACS** (0, 5, 10, 15, 20 µM) for 24 h; Figure S7: ^1^H NMR spectrum of 6-bromo-1,2-anthracene dicarboxylic acid anhydride in CDCl_3_; Figure S8: ^1^H NMR spectrum of **1** in CDCl_3_; Figure S9: ^13^C NMR spectrum of **1** in CDCl_3_; Figure S10: ^1^H NMR spectrum of **2** in CDCl_3_; Figure S11: ^13^C NMR spectrum of **2** in CDCl_3_; Figure S12: ^1^H NMR spectrum of **3** in CDCl_3_; Figure S13: ^13^C NMR spectrum of **3** in CDCl_3_; Figure S14: ^1^H NMR spectrum of **mito-ACS** in CDCl_3_; Figure S15: ^13^C NMR spectrum of **mito-ACS** in CDCl_3_.
