# Supplementary material for: An Anthracene Carboxamide-Based Fluorescent Probe for Rapid and Sensitive Detection of Mitochondrial Hypochlorite in Living Cells
Source: Biosensors (Basel). 2023 Sep 12;13(9):883. doi: 10.3390/bios13090883 (PMC10526414; doi:10.3390/bios13090883)
Supplement: Supplementary file 1 [file biosensors-13-00883-s001.zip › biosensors-2583976-supplementary.pdf]

Supporting Information

An anthracene carboxamide-based fluorescent probe for rapid and sensitive detection of mitochondrial hypochlorite in living cells

Xueling Liu [1, 2], Yali Wang [2, 3], Guangshuai Zhou [2], and Wenzhou Zhang [1, *]

[1]Department of Pharmacy, The Affiliated Cancer Hospital of Zhengzhou University & Henan Cancer Hospital, Zhengzhou 450008, China

[2]School of Pharmaceutical Science and Technology, Health Sciences Platform, Tianjin University, Tianjin 300072, China

[3]Department of Chemistry, College of Pharmacy, North China University of Science and Technology, Tang Shan, 063000, China

***Correspondence:** zlyyzhangwenzhou0551@zzu.edu.cn (W.Z.)

Contents

1. Preparation of reactive oxygen species (ROS) and reactive nitrogen species (RNS)

2. Determination of the detection limit

4. Fluorescence spectra of the probe mito-ACS with and without excessive ClO^-

5. ESI-MS and HPLC data of mito-ACS and the reaction mixture of mito-ACS with NaOCl

6. pH influence of the probe mito-ACS toward NaOCl.

7. Photostability of probe mito-ACS and the oxidation product toward NaOCl.

9. Cytotoxicity assays

10. NMR spectra

1. Preparation of reactive oxygen species (ROS) and reactive nitrogen species (RNS)

All the stocking solutions of ROS/RNS were prepared based on the reported literature [1]**.** The stock hydrogen peroxide (H_2O_2), sodium hypochloride (NaClO), and tert-butyl hydroperoxide (TBHP) solutions were purchased from Sigma-Aldrich. Superoxide anion (O_2^-) solution was prepared by fully dispersing the potassium dioxide in anhydrous DMSO via ultrasonic treatment. Hydroxyl radicals (·OH) and tert-butoxy radical (tBuO·) were prepared by the Fenton reaction, and the molar ratio of $FeSO_4$:H_2O_2 and $FeSO_4$:TBHP was 1:10. Peroxyl radicals (ROO·) were generated from 2,2'-azobis(2-amidinopropane)dihydrochloride. Peroxynitrite ($ONOO^-$) solution was prepared by 3-morpholinosydnonimine hydrochloride (SIN-1). NO· were diluted from the commercially available 2,2'-azobis (2-amindinopropane) dihydrochloride and sodium nitroferricyanide(III) dihydrate (SNP) to ultrapure water.

2. Determination of the detection limit

The detection limit was calculated based on the method reported in the previous literature by the equation as follows:

Detection limit = 3σ/k [2]

where σ is the standard deviation of blank measurement and k is the slope of the equation between fluorescence intensity and the concentrations of NaOCl. We measured the fluorescence intensity of the probe **mito-ACS** without NaOCl for six times to obtain the standard deviation, and the slope k was obtained according to the linear equation of the fluorescence intensity F_{575} with the increasing concentration of NaOCl.

3. The synthesis of starting material

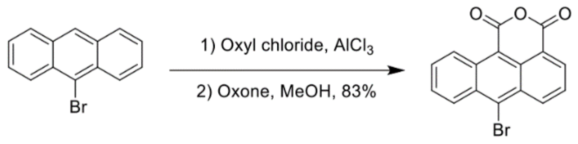

To a 0 ˚C stirred solution of 9-bromoanthracene (3.25 g, 10 mmol) and oxalyl chloride (4.8 mL) in DCM (50 mL), anhydrous $AlCl_3$ (2.13 g) was added. After 2 h, additional DCM (50 mL) and $AlCl_3$ (1.6 g) were added, and stirring was continued for another 2 h at 0 ˚C and then overnight at room temperature. Dilute aqueous HCl (2 M) was added and the orange precipitate was collected by filtration, washed with water, and then digested with 100 mL of 5% NaOH. The solids were washed with water and dried, further treated with oxone (5.6 g, 20 mmol) in MeOH (100 mL), and the mixture was refluxed for 72 h. After cooling, water was added, and the suspension was collected by filtration, washed with water, and dried in air to afford the compound as a brown solid (2.68 g, 83%). The spectroscopic data was in agreement with the data reported in the literature [3]. ^1H NMR (400 MHz, $CDCl_3$): ^1H NMR (400 MHz, $CDCl_3$) δ 9.16 (d, *J* = 8.3 Hz, 1H), 8.60 (dd, *J* = 22.7, 8.9 Hz, 2H), 8.04 (d, *J* = 6.7 Hz, 1H), 7.86 – 7.72 (m, 3H).

4. Fluorescence spectra of the probe mito-ACS with and without excessive ClO⁻

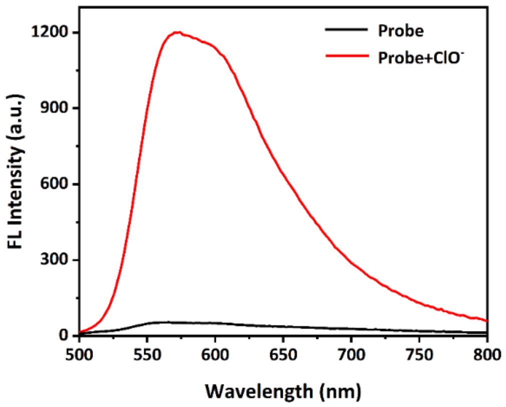

Figure S1. Fluorescence spectra of the probe **mito-ACS** (10 μM) with and without excessive ClO^- (30 μM) in PBS, 10 mM, pH = 7.4, λ_{exc} = 480 nm, slits = 2/2 nm.

5. ESI-MS and HPLC data of mito-ACS and the reaction mixture of mito-ACS with NaOCl

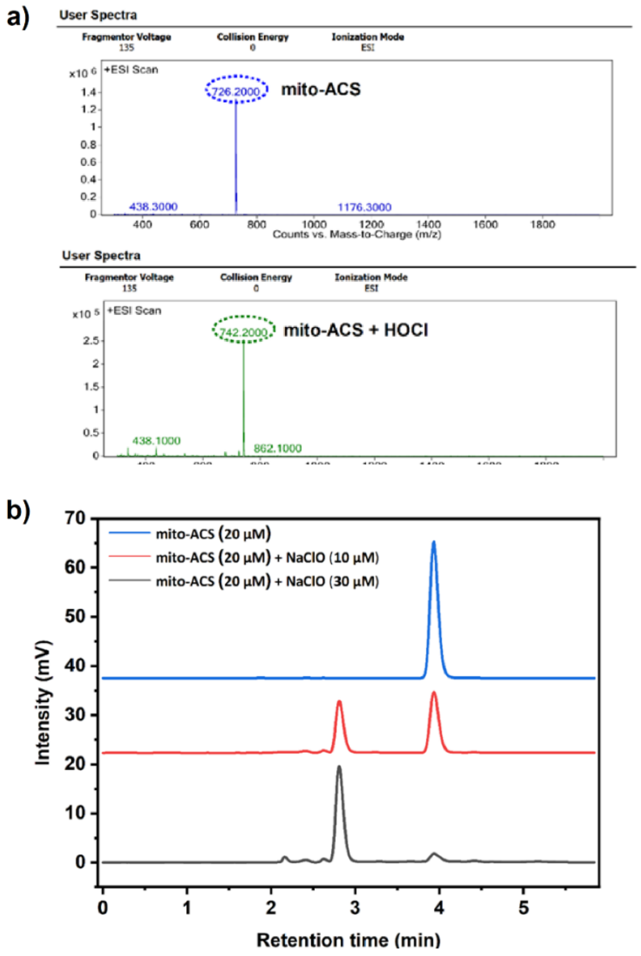

Figure S2. (a) MS-ESI spectra of **mito-ACS** and the reaction mixture of **mito-ACS** with NaOCl;

(b) HPLC analysis of 20 μM **mito-ACS** without or with different amounts of ClO^- (methanol/water solvent mixture (70 : 30, v/v) as the eluent, monitored at 480 nm.

6. pH influence of the probe mito-ACS toward NaOCl.

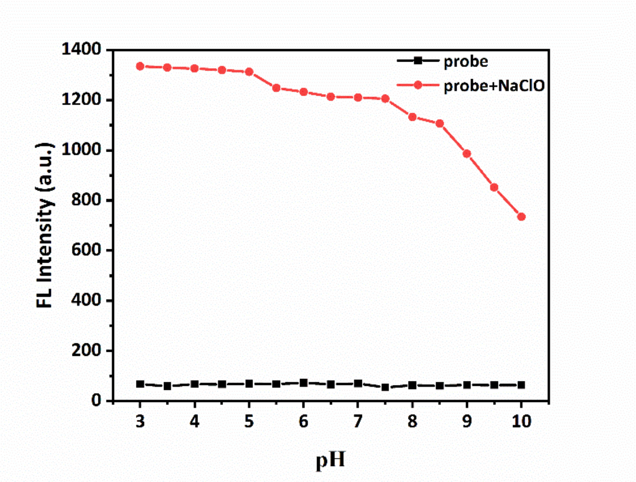

Figure S3. Fluorescence response of **mito-ACS** (10 μM) in the absence and presence of NaOCl (30 μM) at different pH solutions. All data were recorded in different pH buffer solutions (10 mM). λ_{exc} = 480 nm, slits = 2/2 nm.

7. Photostability of probe mito-ACS and the oxidation product toward NaOCl.

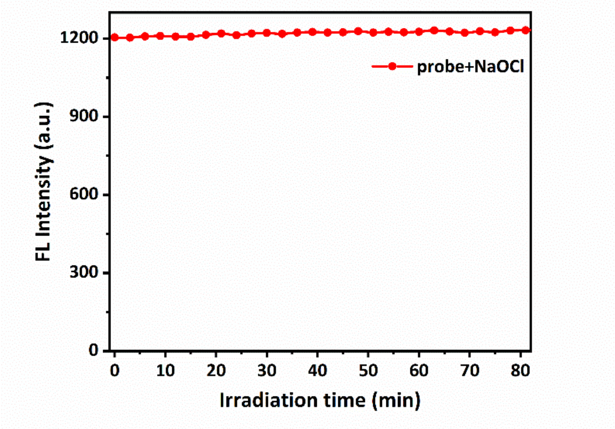

Figure S4. Time-dependent fluorescence intensity changes of **mito-ACS** (10 μM) under the irradiation by a 450w lamp (PBS, 10 mM, pH = 7.4), λ_{ex} = 480 nm, slits = 2/2 nm.

8. Reactivity of probe mito-ACS and the oxidation product toward GSH.

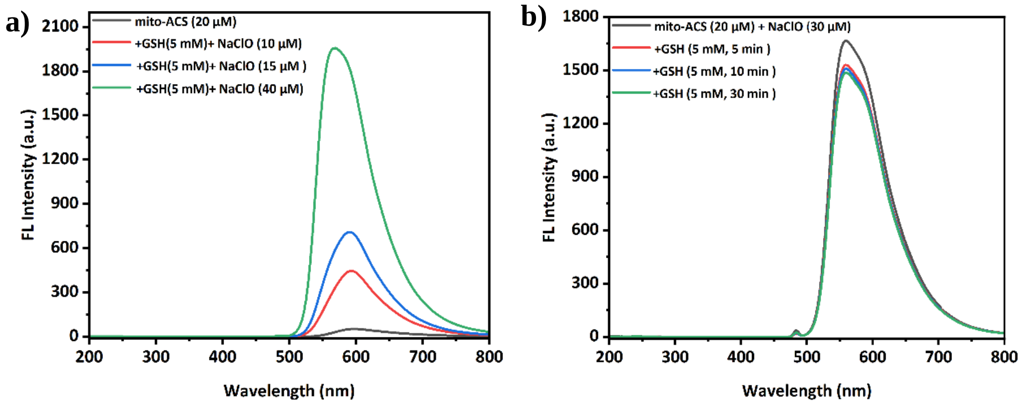

Figure S5. (a) Fluorescence spectra response of **mito-ACS** (20 μM) with GSH (5 mM) in response to different concentrations of ClO^- (10 μM, 15 μM, 40 μM). (b) Fluorescence spectra response of **mito-ACS** (20 μM) with NaClO (30 μM) in response to GSH (5 min, 10 min, 30 min). λ_{exc} = 480 nm, slits = 2/2 nm. (PBS, 10 mM, pH = 7.4)

9. Cytotoxicity assays

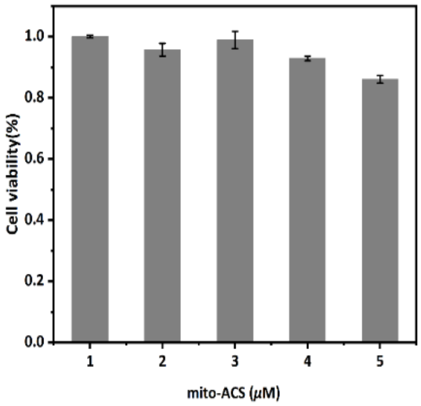

Figure S6. Cell viability of HeLa cells treated with different concentrations of **mito-ACS** (0, 5, 10, 15, 20 μM) for 24 h.

10. NMR spectra

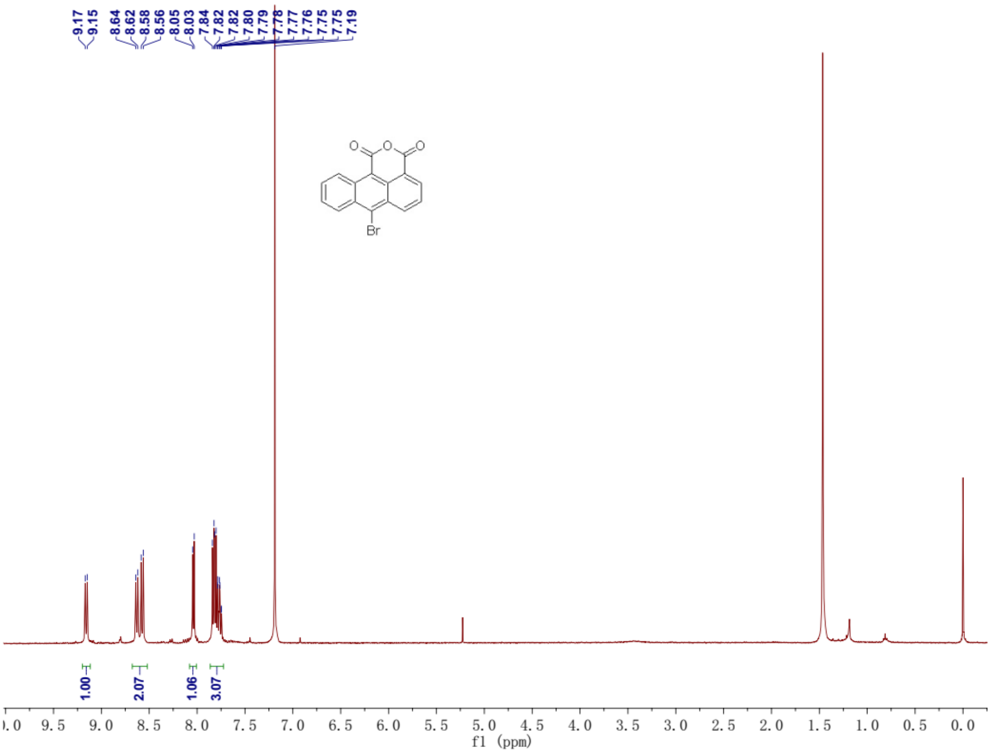

Figure S7. ^{1}H NMR (400 MHz) spectrum of 6-Bromo-1,2-anthracene dicarboxylic acid anhydride in $CDCl_3$.

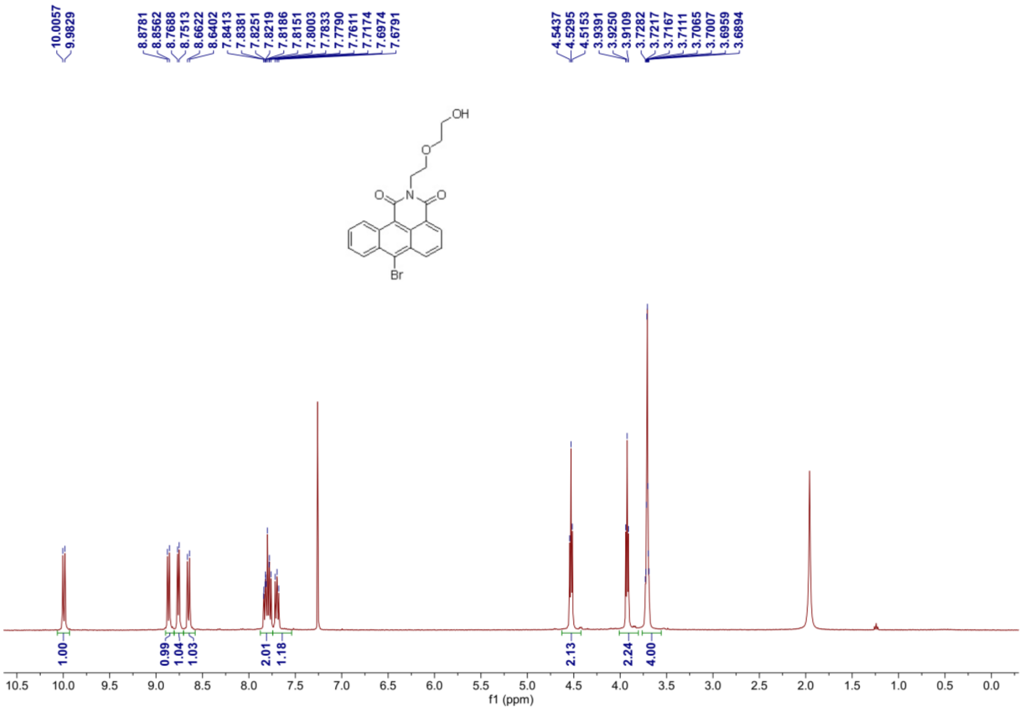

Figure S8. ^{1}H NMR (400 MHz) spectrum of **1** in $CDCl_3$.

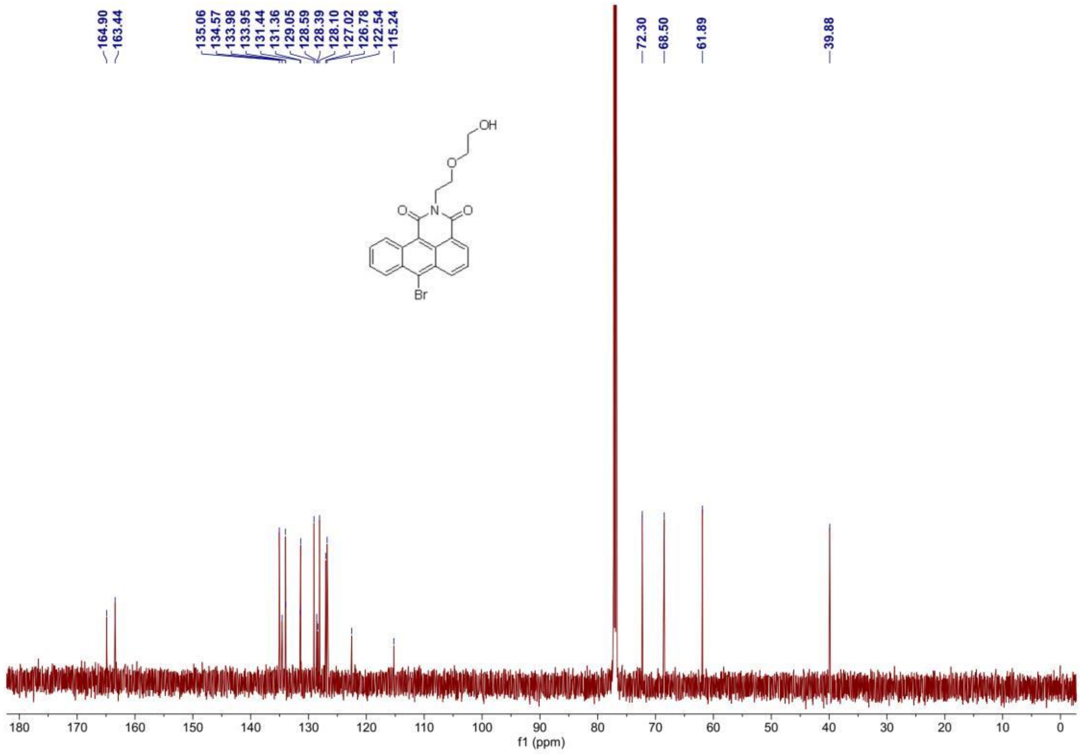

Figure S9. ^{13}C NMR (151 MHz) spectrum of **1** in $CDCl_3$.

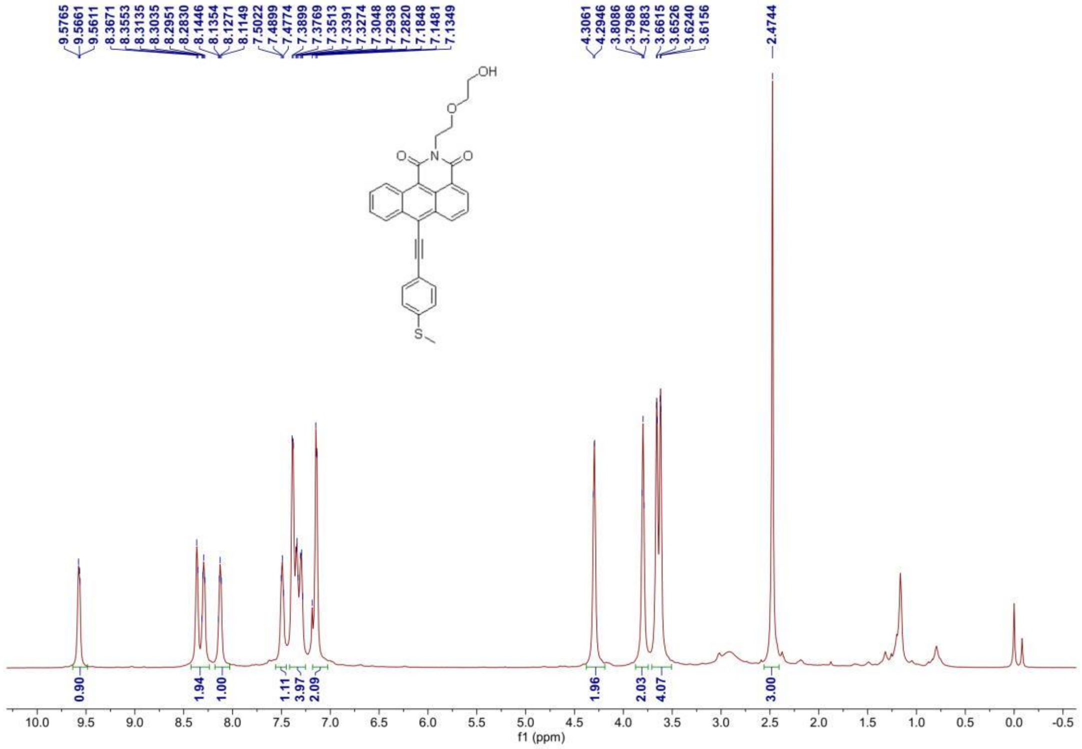

Figure S10. ^{1}H NMR (600 MHz) spectrum of **2** in $CDCl_3$.

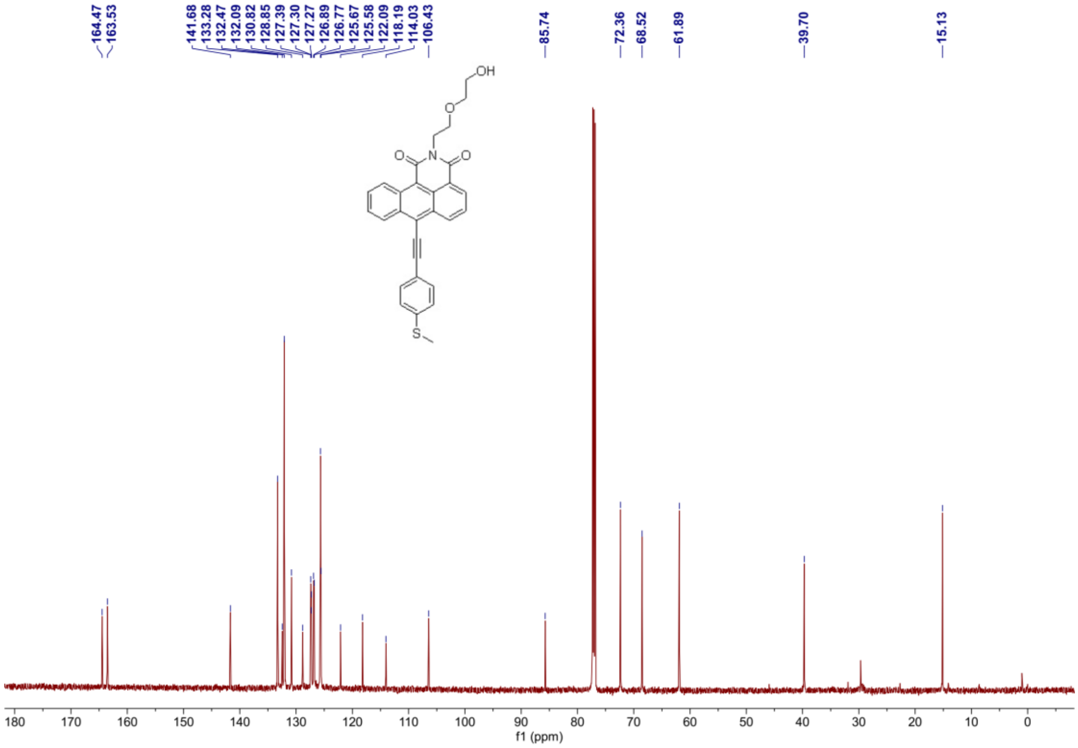

Figure S11. ^{13}C NMR (151 MHz) spectrum of **2** in $CDCl_3$.

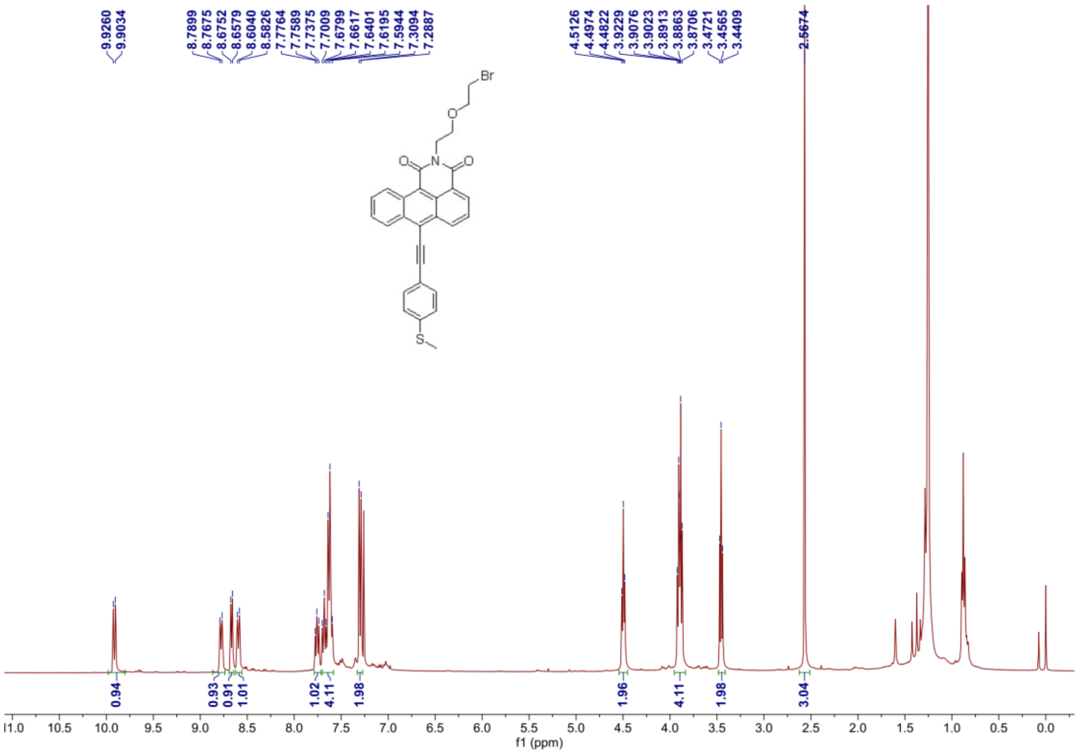

Figure S12. ^{1}H NMR (400 MHz) spectrum of **3** in $CDCl_3$.

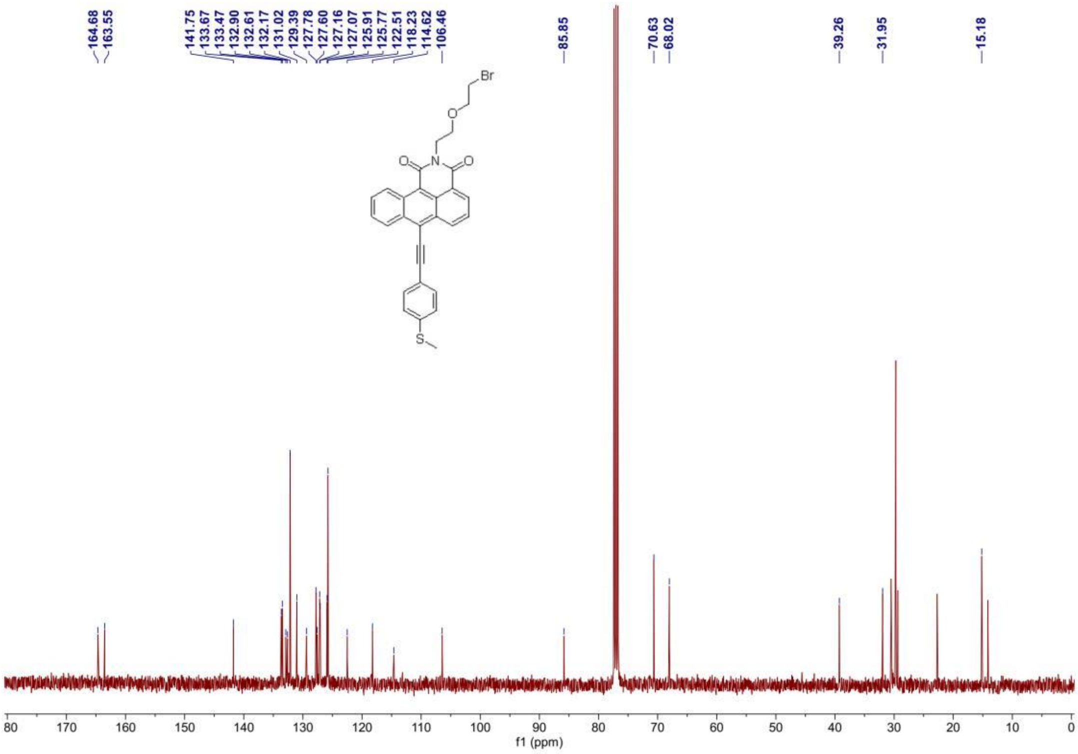

Figure S13. ^{13}C NMR (101 MHz) spectrum of **3** in $CDCl_3$.

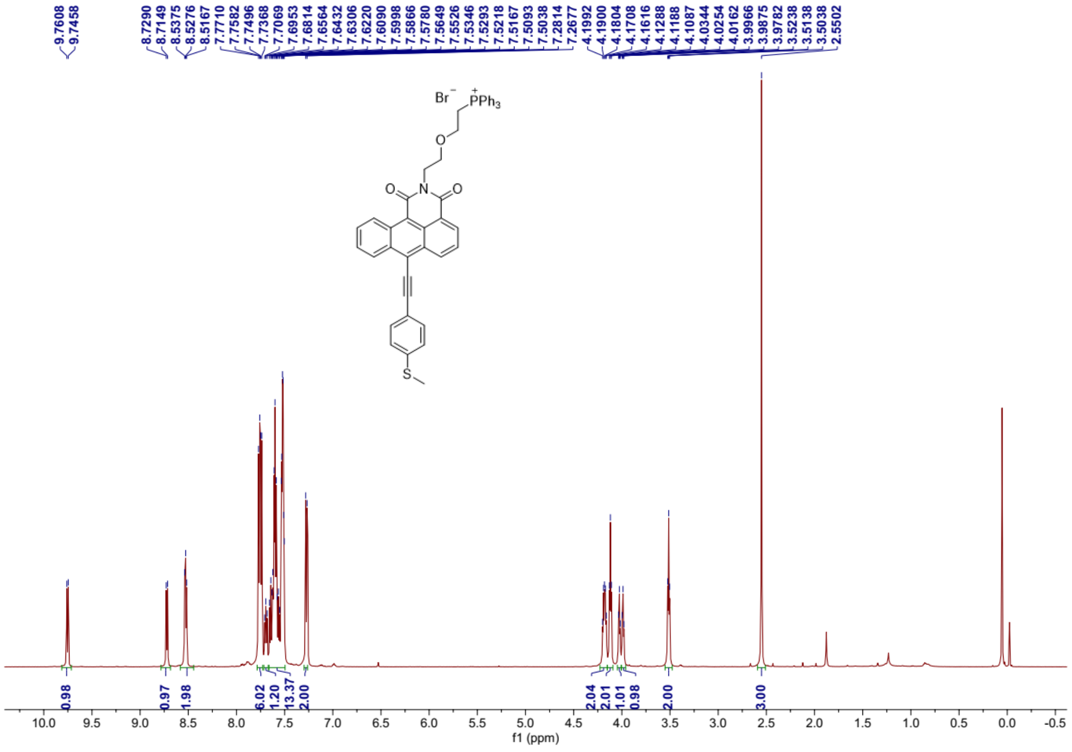

Figure S14. ^{1}H NMR (400 MHz) spectrum of **mito-ACS** in $CDCl_3$.

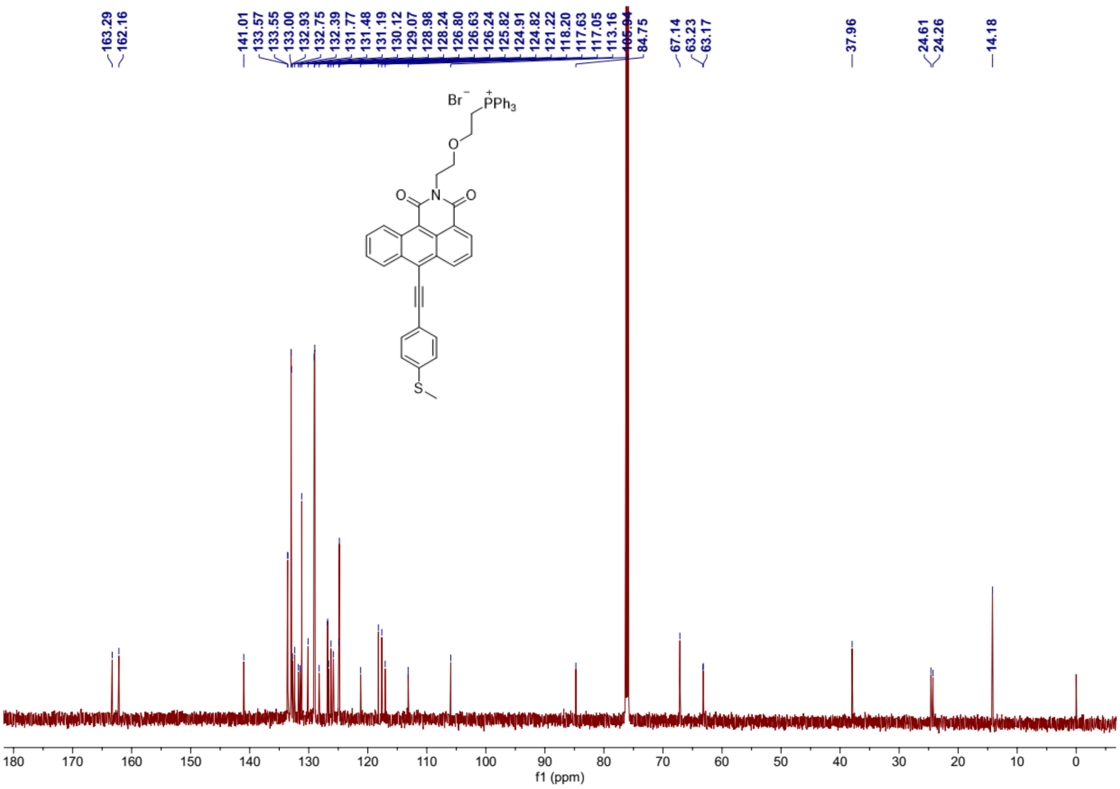

Figure S15. ^{13}C NMR (101 MHz) spectrum of **mito-ACS** in $CDCl_3$.

1. Zhu, B.; Gao, C.; Zhao, Y.; Liu, C.; Li, Y.; Wei, Q.; Ma, Z.; Du, B.; Zhang, X., A 4-hydroxynaphthalimide-derived ratiometric fluorescent chemodosimeter for imaging palladium in living cells. *Chem. Commun.* **2011,** 47, (30), 8656-8658.
2. Zeng, L.; Xia, T.; Hu, W.; Chen, S.; Chi, S.; Lei, Y.; Liu, Z., Visualizing the Regulation of Hydroxyl Radical Level by Superoxide Dismutase via a Specific Molecular Probe. *Anal Chem* **2018,** 90, (2), 1317-1324.
3. Xu, J.; Niu, G.; Wei, X.; Lan, M.; Zeng, L.; Kinsella, J. M.; Sheng, R., A family of multi-color anthracene carboxyimides: Synthesis, spectroscopic properties, solvatochromic fluorescence and bio-imaging application. *Dyes Pig.* **2017,** 139, 166-173.
